# Shelters Reflect but Cannot Solve Underlying Problems with Relinquished and Stray Animals—A Retrospective Study of Dogs and Cats Entering and Leaving Shelters in Denmark from 2004 to 2017

**DOI:** 10.3390/ani9100765

**Published:** 2019-10-05

**Authors:** Peter Sandøe, Janne B.H. Jensen, Frank Jensen, Søren Saxmose Nielsen

**Affiliations:** 1 Department of Veterinary and Animal Sciences; University of Copenhagen, 1850 Frederiksberg C Copenhagen, Denmarksaxmose@sund.ku.dk (S.S.N.); 2 Department of Food and Resource Economics; University of Copenhagen, 1958 Frederiksberg C Copenhagen, Denmark; fje@ifro.ku.dk

**Keywords:** animal shelter, canine, Denmark, economics, euthanasia, feline, intake, live release rate, regulation

## Abstract

**Simple Summary:**

Large numbers of dogs and cats end up as stray and homeless animals, and they are often viewed as a problem. Animal protection non-governmental organizations (NGOs), particularly in the Western world, have established shelters to deal with this issue. Their ideal goal is to rehome stray and abandoned dogs and cats to avoid them living difficult lives as strays or being euthanised. The current study assessed the number of cats and dogs entering and leaving shelters in Denmark over a 14-year period and aimed to determine how well this goal has been achieved. Denmark is interesting because there are no stray dogs, while cats to a large extent roam freely. In the case of dogs, there was a relatively low and slight decreasing intake and a low euthanasia rate during the period. From the start there was a higher intake and a higher euthanasia rate for cats, and during the period shelters had a 250% increase in the intake of cats. Although a larger number of cats were adopted, the proportion of shelter cats that were euthanised increased from 15% to 29%. Increased shelter capacity by itself cannot solve the problem with stray cats.

**Abstract:**

Data covering about 90% of the estimated intake of dogs and cats to Danish shelters from 2004 to 2017 were used to study the effects of tight control of dogs and of efforts to increase shelter services for unwanted or stray cats. During the period, there was a low and decreasing intake of dogs, while the annual proportion of euthanised dogs increased from 6% to 10%. The number of cats entering shelters increased by about 250%, while the annual proportion of euthanised cats increased from 15% to about 29%. At the same time, there seemed to be a decrease in the population of stray cats. The major increase in cat intake may be due to animal protection non-governmental organizations (NGOs) making it easier to relinquish cats into shelters. Dog shelters can successfully handle surplus animals because dogs are well controlled by owners and are tightly regulated. Cats are more difficult to confine, are often allowed to roam freely and are less regulated. Therefore, cat shelters cannot solve the problem of surplus cats on their own. It is argued that an economic analysis may serve as a point of departure for a discussion on better policy making for NGOs in charge of shelters.

## 1. Introduction

Unwanted stray and homeless dogs and cats constitute a considerable problem in large parts of the world. Traditionally, the problem was thought to concern human health and safety primarily, and the main response was to euthanise these animals in large numbers [1]. Increasingly over the last three to four decades, the perception of this problem has changed in most Western countries, with a focus on avoiding euthanasia of stray and unowned dogs and cats. As a result, animal protection non-governmental organizations (NGOs)—and in some countries also public authorities—have invested in programmes to increase adoptions from shelters with the aim of minimising the need to kill these animals and instead find new homes for them. Undoubtedly, this change of focus has saved the lives of many millions of healthy dogs and cats. There has also been an increasing focus on the need for microchips, collars and identity (ID) tags to be able to reunite owners with dogs and cats that have gone astray [2]. In addition, publicly sponsored neutering programmes has been considered as a means to prevent the birth of unwanted puppies and kittens, thereby limiting shelter intake [3,4].

However, there have been growing concerns about the extent to which shelters serve to either solve or exacerbate the underlying problems relating to unwanted or stray dogs and cats [5], i.e., that these animals live in poor conditions, reproduce in an uncontrolled manner, are viewed as a nuisance, and are euthanised in large numbers. These concerns go hand-in-hand with a growing effort to document the effects of shelters with true numbers of animals entering and leaving shelters, where many early studies on the subject seemed to have been based on rough and very diverse estimates [1,6]. Thus, there is a widely recognised need for more knowledge on factors affecting shelter intake and adoption rates, and a recent review noted that most studies are only conducted at a single point in time and at one location, making it difficult to document spatial and temporal changes [5]. The current study looks at the role of shelters in managing the number of unwanted and stray animals in Denmark. This country is an interesting case because there is a major contrast between how dogs and cats are kept and regulated in Denmark.

About 20% of Danish households own a dog and a similar number of households have one or more cats [7,8]. Legal regulations require that all dogs must be registered before the age of 8 weeks and there seems to be very high compliance. At the same time, people are required to keep their dogs confined or under strict control typically behind fences in gardens or on a leash when walking in urban areas [9]. There is no requirement to register cats, and even though people are obliged in principle to keep their cat on their own property, this legislation is not really enforced and cats are often left to roam freely [7]. According to [10] 71% of households with privately owned Danish cats allow their cats to roam all the time or on a regular basis. Therefore, some significant differences can be expected between these two species in terms of problems with stray animals.

Some Danish owners may want to relinquish or are no longer able to look after their dogs or cats. They can either sell them, give them away, go to a vet and have them euthanised, or take them to a shelter (some of which take the animals in for free while others charge a handling fee). There are no public shelters in Denmark—they are all owned by NGOs or private individuals. Furthermore, the way that shelters and other larger facilities keep dogs or cats is tightly regulated [11]. It is also possible that owners may abandon their dogs and cats so that they end up becoming strays or homeless animals. 

In Denmark, all stray dogs are picked up, typically by the police or a private emergency service with which the authorithies have a contract, and are placed in some form of custody. Then it is advertised that the dog has been found, and the owner can get the dog back by paying the costs incurred. If no owner has reacted within three days the dog may, according to the Danish dog law [9], be euthanised or handed in to a shelter. Consequently, there are no street dogs in this country. 

Cats, on the other hand, have always lived in large numbers as strays in Denmark. Since cats are not covered by a law comparable to the Danish dog law, stray cats are not picked up by the police or any other people working in an official capacity. Instead stray cats are picked up by or handed into various NGOs. 

Some of the cats are unsocialised cats, which are of no relevance to the current study. As a matter of policy the main Danish NGOs do not allow these cats to enter shelters because they cannot become companion animals and because it is viewed as cruel to keep them confined (although there are some borderline cases and some unsocialised cats that enter shelters by mistake). Instead unsocialized cats are either euthanized or re-released as part of the trap-neuter-and-return programmes. 

Socialised stray cats come from a number of sources. They may have been born on a farm and wandered off, they may have lived as companion animals and gone astray (and if they have not been earmarked or microchipped, which it is not legally required for a cat owner to do in Denmark, then it can be difficult to find the original owner), or they may have been abandoned by their original owners. Furthermore, it is common for cats to be left at shelters anonymously in boxes, bags, or cages.

Although there are a number of studies focusing on the effects of shelters measured in terms of the number of animals leaving the shelters alive relative to the total number of animals passing through in a given time period of time, to our knowledge, we are the first to study the flow of animals in and out of shelters over a longer period of time, i.e., 13 years. Based on our data we aim to address the following two questions: (1) What happens to intake, return to owner, adoption and euthanasia over the period when dogs are tightly regulated? (2) What happens when there is no regulation of cats, shelter capacity is increased and it becomes easier to surrender a cat to a shelter? 

The principal conclusions of the paper are: (1) when dogs are generally kept in a controlled way and are tightly regulated as is the case in Denmark, the problem of unwanted and stray dogs is limited, and shelters can handle this well; (2) when there is a large surplus of stray cats, efforts to make it easier to move cats into shelters will result in both a large increase in stray cats being adopted and a major increase in the number of shelter cats being euthanised. 

## 2. Materials and Methods 

### 2.1. Identification and Inclusion of Shelters

Danish shelters were identified via a search in Google^®^ using the search terms “dyreinternater i Danmark” (“animal shelters in Denmark”) and “danske dyreorganisationer” (“Danish animal organisations”). Furthermore, the Danish Central Business Register (www.virk.dk) was searched, and the Danish Veterinary and Food Administration (Glostrup, Denmark) was asked to provide a list of shelters. The authorities have this information, at least for shelters with dogs, as according to Danish law premises with more than four adult dogs (>18 weeks old) are considered professional entities and must be recorded as such [11].

The shelters identified via these sources were listed and it was then assessed whether they were still active or had ceased to operate. The managers of shelters that were active at the time of the study were invited to contribute to the research, which was described in the invitation. Two reminders were sent to non-responders. Participants were assured anonymity in a written statement (see Supplementary text S1).

### 2.2. Data Manipulation 

The participating shelters were asked only to submit data on the intake of dogs and of socialised, potentially adoptable, cats. Participating organisations provided intake and outcome data summarised on a yearly basis for the study period. These data were arranged under the categories: (a) owner relinquished including returns (animals adopted from the shelter but later returned to the shelter by the new owner); (b) strays (animals handed in by authorities, professionals and private citizens stating that they had no relation to the animal, and in the case of cats this included only socialised and not feral animals); (c) rescues (animals obtained following cases of neglect); (d) others (including animals born in the shelter, transfers between shelters or from veterinary clinics, and those dumped outside the shelter). The output from the shelters included the categories: (i) rehomed (where the animal was adopted by a private individual); (ii) returned to owner (typically strays with identification or that were located by their owner); (iii) euthanised; iv) dead; v) others (including transfers between shelters and lost and stolen animals). Unlike shelters in some other countries, Danish shelters do not perform requested euthanasia and, therefore, this is not part of the output. Shelter managers were interviewed about their definitions of data variables to resolve any issues caused by discrepancies in their data-recording practices. Major inconsistencies between intake and outcome data were resolved with the shelter staff.

The data were tabulated for each species, year, and input or output category. The live release rates (LRR) were calculated as the proportion of all live output from all categories of outcome including euthanasia and death: (n_rehomed_ + n_returned to owner_ + n_others_)/n_left shelter dead or alive_) [12] for each year. The total intake and LRR were plotted as a function of year. Furthermore, the proportion of each category of intake and output for each species was plotted over time.

### 2.3. Completeness Assessment

Not all participating shelters provided data for all years. The number of omitted cases were interpolated from the years they did provide data, based on the average intake and outcome in the years included, which was deemed reasonable based on visual inspection of the data. Furthermore, the number of animals handled at shelters that did not contribute data was estimated based on: (a) a survey of their website, or (b) personal contact with people linked to the shelter requesting a best estimate of the population they handled. This information was used as the basis for a crude estimate of our survey coverage. 

### 2.4. Proxy Measure of the Population of Stray and Homeless Cats

As a proxy measure of the population of homeless and stray cats, we obtained annual summarised records of cats hit by vehicles. We obtained this information from one of the major organisations, which has a hotline that people can use to report animals injured in traffic. The data were available for 2012 to 2018, and were plotted alongside the annual intake data.

### 2.5. Information from Non-Governmental Organizations (NGOs) in Charge of Danish Shelters Handling Cats

In light of our finding of a dramatic increase in the intake of cats into Danish shelters during the period studied, an email was sent to four senior contacts who worked or had previously worked with the four main NGOs in charge of Danish shelters with a large intake of cats. The email contained questions about this development (see Supplementary text S2).

## 3. Results

We identified 20 organisations that managed 52 shelters in total, with 37 (71%) of the shelters providing data for the study. Of these, 19 managed only cats, one managed only dogs, and the remainder managed both species. One dog shelter did not provide data for 1 year, thus excluding an estimated 109 dogs. Five cat shelters did not provide data for 2 to 9 years, excluding an estimated 13,600 cats. Furthermore, among the shelters that did not provide data for the study, websites and personal contacts of the managers suggested that these managed approximately 1429 cats and 99 dogs in 2017. Therefore, with approximately 11,240 cats included and approximately 1429 cats not included in 2017, the best estimate of completeness was 89% for cats. The intake of dogs was around 1553 in 2017, so with approximately 99 dogs not included this means that the completeness was approximately 94% of the estimated total number of animals at the national shelters.

**Figure 1:**
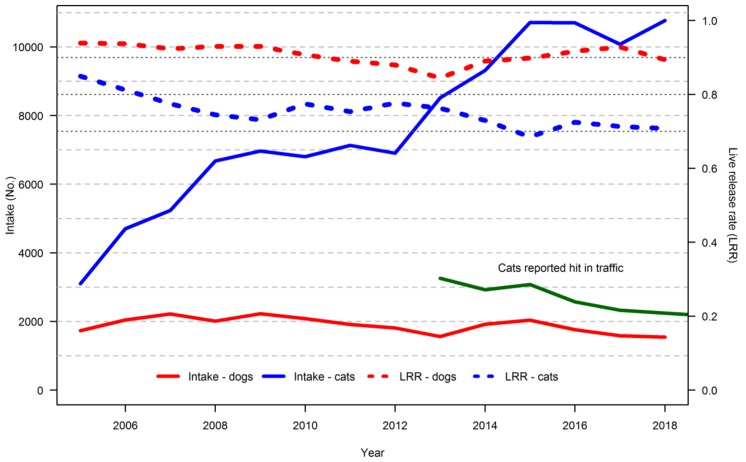
Intake numbers (full red and blue lines) and live release rates (dotted lines) for cats and dogs, summarised for 37 shelters in Denmark. Annual summarised records of cats hit by vehicles from one of the major organisations were also obtained (green line).

The intake of dogs decreased during the study period from 2004 to 2017 from around 2000 to 1550 annually, while the intake of cats increased from 3100 in 2004 to more than 11,000 in 2017 (Figure 1). The live release rates for both species decreased from around 0.94 in 2004 to 0.90 in 2017 for dogs, and from 0.85 in 2004 to 0.71 in 2017 for cats (Figure 1). The number of cats reported hit by vehicles decreased from 3259 in 2012 to 2166 in 2018 (Figure 1), corresponding to a decrease of 33%. 

**Figure 2:**
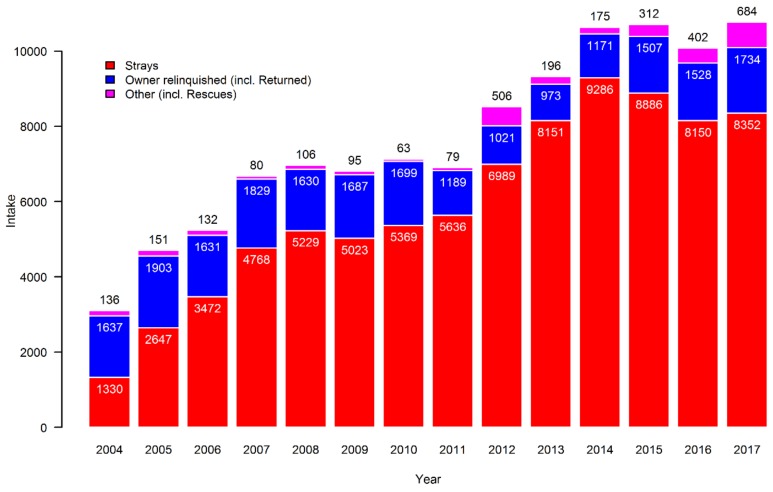
Sources of cats in the Danish shelters over the study period from 2004 to 2017.

There was a major change in the sourcing of cats for shelters during the timespan of the study (Figure 2). At the beginning of the period, roughly half were owner relinquished and the other half were strays. While the number of owner-relinquished cats was relatively stable, there was a dramatic increase in the number of stray cats entering shelters over the period, so that in recent years only 10%–15% of cats entering shelters were owner-relinquished, whereas about 80% were strays.

**Figure 3:**
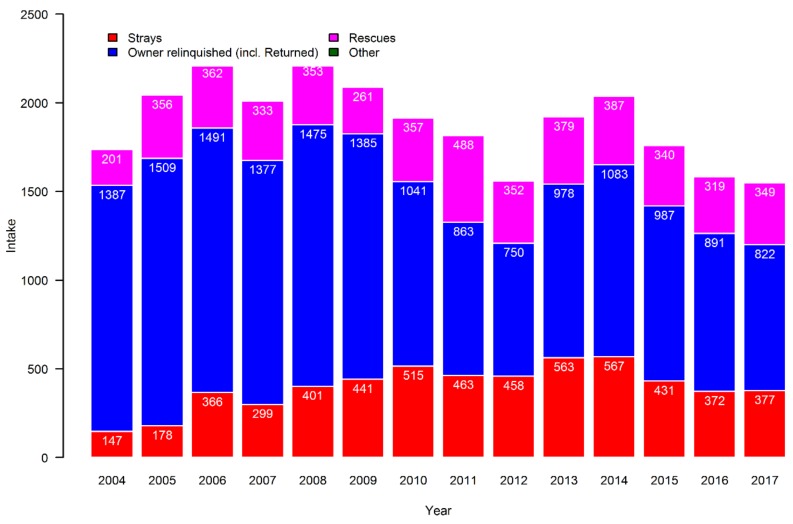
Sources of dogs in the Danish shelters over the study period from 2004 to 2017.

Unlike for cats, as can be seen from Figure 3, the majority of dogs entering Danish shelters during the period were owner-relinquished, while the groups of strays and rescued dogs are of equal size. Over time, the number of owner-relinquished dogs dropped, while there was an increase in the number of stray dogs. Returns only accounted for around 1% annually, whereas rescues varied between 12% and 27% of the yearly intake, with no obvious patterns.

**Figure 4:**
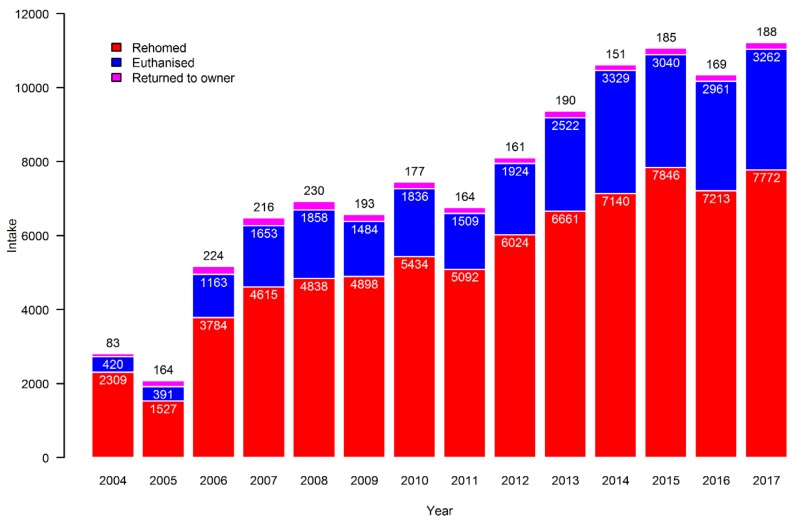
The final outcomes for cats placed in Danish shelters from 2004 to 2017. The categories “Dead” and “Other” contained only 30 and 5 cats, respectively, and are therefore not evident.

As can be seen from Figure 4, most cats that entered the Danish shelters were either rehomed or euthanised. The absolute number of cats rehomed more than tripled over the period, but the relative proportion of cats that were euthanised almost doubled from around 15% to 29% of the yearly output. 

**Figure 5:**
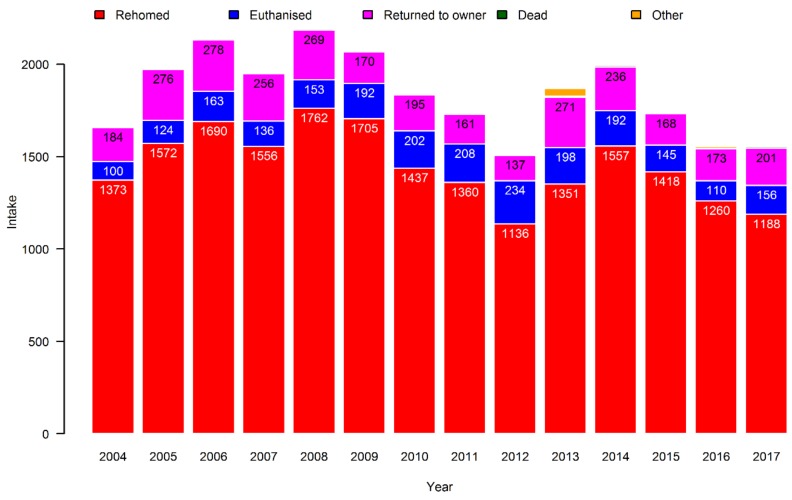
The final outcomes for dogs placed in Danish shelters from 2004 to 2017. The category “Dead” contained only three dogs, and is therefore barely visible.

As can be seen from Figure 5, the vast majority of dogs entering Danish shelters were rehomed. A significant proportion (between 8% and 15% of the yearly output) of dogs were returned to the owner, while there was an increase in the proportion (from 6% to around 10% of the yearly output) that were euthanised. 

All four senior contacts who worked or had previously worked with the four main NGOs in charge of Danish shelters dealing with cats responded to our email. Three of the respondents still worked with the respective NGO. The responses reflected differences in how the NGOs operated, but they agreed on the overall story:

Ten years prior to the start of the period studied, approximately 50% of cats entering shelters were euthanised. After this, some Danish NGOs with an interest in cats put more focus on how they could become better at rescuing and rehoming unwanted and stray cats. This led to more potentially adoptable cats entering shelters and to more cats being adopted. Around the start of the study period, a growing collaboration developed among the NGOs running shelters and there was an expansion of capacity, making it easier to pass a cat on to a shelter. Around 2011, the largest NGO established a national hotline that people could call and ask to have cats in distress picked up for free, but through the hotline people could also get information about how to relinquish stray cats to a shelter.

The email from author Peter Sandøe to the NGOs as well as their responses (in an anonymised version) are available as an English translation in the supplementary files (Supplementary text S2).

## 4. Discussion

### 4.1. Discussion of Main Findings

The first of our findings that should be discussed is the low and slightly decreasing intake of dogs in Danish shelters during the period. At the beginning of the period, about two thirds of the dogs were relinquished by their owners, and at the end of the period this number decreased to around half of the dogs. Dogs that are not ill, old or have serious behavioural problems should be easy to adopt since there is a large demand for dogs.

The reasons for the simultaneous decrease in the intake of relinquished dogs and the live release rate seem to be connected. In recent years, social media has provided new and cheaper options to people who no longer want or are able to own a dog to pass the animals on to new prospective owners. They may even sell their dogs to make a profit. The dogs that entered shelters in the second half of the period studied may have been more difficult to pass on (i.e., they were more likely to be ill, old or have behavioural problems). This would have resulted in a greater proportion of these dogs being euthanised rather than adopted, despite a reasonably constant and small absolute number of shelter dogs being euthanised overall.

Most of the dogs that enter Danish shelters are rehomed. In the case of dogs then, it seems that the ideal rationale of shelters works out; thanks to the shelters, stray and unwanted dogs in difficult situations are taken care of, and most adoptable dogs are being adopted.

The situation for cats in Danish shelters is very different. Over the study period, there was a 250% increase in the number of cats entering shelters, from around 3000 per year to around 11,000 per year. We have no reason to believe that the number of stray cats increased during this period; on the contrary, the number of stray cats may have decreased as suggested by the lower number reported above as being hit by vehicles over a period when traffic increased. Thus, according to the Danish Road Directorate the amount of road traffic in Denmark increased 14% from 2010 to 2018 [13]. These numbers include both owned and stray cats but since we know that the number of owned cats increased during the period [7], this finding gives us a good reason to believe that the number of strays has decreased. This raises the question of why there was a higher intake.

Stray cats may be seen as a problem by two different groups of people based on two opposing views: some people view them as a nuisance because they make noise, leave their excrement in sandboxes and gardens, or for some other reasons (21% according to [10]). Other people may feel pity for them and want them to have a better and safer life by being rehomed via a shelter. People with these contrasting sentiments may want to change the ‘stray’ status of these cats. They may be willing to pay for this to happen, but they will typically want it to happen at as low a cost as possible.

To understand what drives the higher intake of stray cats into shelters, it is important to note that there may be other possible options for people who view unwanted or stray cats as a problem:

- They may choose to live with the problem of stray cats in their vicinity.
- They may take the cats to the vet, who may put them down (at a price of around 110 EUR or more)—if it is a cat that is owned but unwanted, this can be done at the request of the owner, and if it is without an owner, the vet may put it down if permitted by the municipality (a permission that is almost always given).
- If they have a hunting license, they may also choose to kill their own unwanted cats, or ask someone with a hunting license to do so.
- They may also kill or have someone kill the cats illegally.

Each of these options comes at a cost, emotional and/or financial. When it becomes easier to surrender a cat to a shelter, as it seems to have become during the period studied, it means that more people are likely to prefer that option to one of the aforementioned options. Thus, those who wished to get rid of one or more cats for one reason or another may have been tempted by the easy access to shelters. Those who were bothered by the cats may have seen it as an easy solution, while those who felt pity for the cats may have used the shelter option in the hope that a home could be found for the cats. 

Those who set up and run shelters may have several motives for doing so: one clear motive is to cater to the wishes of the group of people mentioned above who feel pity for homeless or stray cats. These are the people who typically support animal protection NGOs by becoming members and giving various forms of donation. Of course, the NGOs typically also care about dogs and cats and want to help them. The goal is for fewer dogs and cats to experience difficult lives as strays, and for fewer healthy and potentially adoptable dogs and cats to be euthanised.

The development of cat intake observed can in part be explained by one of the organisations changing their surrender procedure. Previously, diseased cats would not be included in the shelter statistics since they were taken directly to a veterinary practitioner for potential euthanasia and, therefore, never entered the shelter. After this change in procedure, the cats typically went through a shelter (Jens Jokumsen, personal communication), so some, but in our view most likely only a part of the higher intake, may be a reflection of this change.

During the same period, an effort was made by the animal protection NGOs running the shelters to increase the adoption rate of cats from shelters primarily by means of social media. As a result of this, the yearly number of shelter cats being rehomed more than tripled during the period. This increase in demand for shelter cats was, apparently, insufficient to keep up with the supply. The proportion of euthanised cats nearly doubled over the period, from around 15% to 29%.

This increase may, as already indicated, to some extent reflect changed management practices, including better screening of shelter cats for serious diseases such as feline immunodeficiency virus (FIV) and feline leukemia (FeLV). However, it may also to some extent be explained in terms of the need to euthanise surplus animals. It should be stressed that our study does not deliver data which can document the relative role of these two explanations of the increase in euthanasia of cats in Danish shelters.

### 4.2. Discussion of Findings from an Economic Market Perspective

Following our analysis above, we believe it could be useful to discuss dogs and cats in shelters from an economic market perspective [14]. It is possible to distinguish between an intake (input) and a release (output) market. 

For the input market, a supply curve can be identified capturing the number of dogs and cats entering shelters. The supply of dogs and cats will decrease with an increase in the cost of bringing animals to shelters. The costs capture a number of factors, including the inconvenience of bringing animals to shelters and driving distance to nearest shelter. A demand curve can also be identified for the input market, and this curve represents the number of animals that shelters take in. If shelters take in all cats and dogs delivered at a certain cost then the demand curve will be a horizontal line. An equilibrium on the input market requires that supply equals demand, and an equilibrium cost on the input market can be identified based on this. 

A supply curve can also be identified on the output market, and if all dogs and cats brought in are rehomed then this curve will be a vertical line equal to the amount of animals entering shelters. A demand curve for dogs and cats on the output market can also be identified and the quantity demanded will increase if the price that shelters require for animals decreases. When supply equals demand on the output market, there will be an equilibrium price for dogs and cats rehomed from shelters. 

For dogs, as we have seen, a high proportion of animals are rehomed from shelters. Furthermore, the supply of dogs on the input market is low and the demand on the output market is high. It matters here that shelter dogs are much cheaper (400 EUR or less) than other dogs on the market, as in Denmark, dogs typically cost between 700 and 2000 EUR, depending on breed. This implies that we are close to an equilibrium on both the input and output market. Thus, from an economic perspective, the markets for shelter dogs are working well. 

However, a lower proportion of cats are rehomed and this seems to be the combined effect of excess supply on both the input and the output market. As discussed above, the emotional and financial price of taking a cat to a shelter has decreased, while the price of adopting a cat from a shelter has been constant. This is typically around 140 EUR, which includes vaccination, neutering, and ear-marking or microchipping. While this price is by no means high, many people acquire cats without paying for them [7]. Even though it has been found that 86% of privately owned cats in Denmark are neutered [7], there still seems to be a large supply of kittens from private homes. In addition, people can often acquire them within their own neighbourhood, whereas they may have to go some distance to a shelter. So despite a considerable market for companion cats, people acquire them from other sources in such numbers that not all shelter cats can be sold. 

To solve this problem, two economic incentives could be imagined: (i) a subsidy to buy cats from shelters; (ii) a tax on bringing cats to shelters. Solution (i) will reduce the cost of obtaining cats from shelters and thereby increase the quantity demanded. The cost of bringing cats to shelters increases with solution (ii), suggesting that the input supplied will be lower. However, a problem with solution (i) is that more people may want to obtain cats from shelters with the consequence that the demand from other supply sources may decrease, while people may try to get rid of cats in other ways with solution (ii). While both a tax and a subsidy would work well from a purely economic perspective, other problems with the two policies may arise, as we have seen. A more advanced economic analysis could try to analyse problems as externalities [15].

Apart from being able to reclaim some of the costs incurred, a motivation for charging a substantial price for shelter cats seems to be that the shelter will ensure that prospective owners are sincere in their wish to acquire a cat and will therefore end up being good and responsible owners. However, there is some evidence from the literature that lowering the price of cats from shelters will not lower the motivation of prospective owners [16].

Overall, the “shelter economy” seems to function less well for cats than for dogs; many more cats are taken into shelters (acquired) than are adopted (sold). Even though a large number of cats are taken care of, a growing number of these cats end up being euthanised. Furthermore, since there may be too many cats relative to the demand, it is possible that the problem of surplus cats is not really being solved, but instead just shifts from one group of cats to another—so that more cats are adopted out of shelters, while fewer are adopted in a more direct way.

By promoting adoptions from shelters, the relevant NGOs may also indirectly create a greater demand for cats. However, there is a danger that a side effect of this greater demand is that more people acquire cats from other sources without having them neutered, microchipped or ear-marked. As a result, the problem of stray cats may actually increase even more. We recommend that more structured economic planning should go into developing strategies for shelters. For an example of an economic approach to the issue, see [17].

### 4.3. Comparison with Findings from Other Countries

Globally, there have been a small number of studies with time series that are comparable to ours. A study of shelters in the USA recorded and compared the intake and output of dogs and cats in Metro Denver, CO, between 1989 and 2010 [18]. The authors found that the intake of dogs and cats was of a roughly equal size, but with more dogs than cats, unlike in our study where the intake of dogs was much lower than the intake of cats. This is likely to reflect the much stricter regulations on keeping dogs in Denmark compared to the USA. The intake of dogs declined over the years studied, as did the rate of euthanasia, while there was an increase in cat intake and a slight increase in the euthanasia rate for cats since 2000. A study from two counties in California [4] covering the period from 1990 to 2005/6 saw a similar development in the intake of dogs as that seen in Denver, but with a decrease in the cat intake, which the authors explained as resulting from the municipal spay/neuter voucher programme for cats. A group with some of the same authors as the Denver study [19] also performed a comprehensive study of animal shelter intake and outcome data for dogs and cats in Colorado covering over 90% of the intake from 2000 to 2007. They reported a decrease in the intake of dogs as well as a low and stable euthanasia rate, and an increase in the intake of cats, with an increasing euthanasia rate for this species. In line with this, an earlier study of animal care and control agencies in Ohio compared figures from 1996 with those from 2004 and found that the intake of dogs decreased as did the euthanasia rate, although this remained at a high level (from 61% to 46%), and while there was an increase in the cat intake, the rate of euthanasia fell, although it was still higher than for dogs (from 72% to 69%) [20]. A study of Michigan shelters in 2003 found the euthanasia rate for dogs to be 40% and for cats 57% [6]. Earlier studies from the USA also found higher euthanasia rates for cats than for dogs [1,21]. In addition, two Australian studies [22,23] found high euthanasia rates (65% and 74%) for cats. Another Australian study of dog intake and outcome data in three metropolitan shelters in 2001 and 2002 found a euthanasia rate for dogs of 32% [24]. 

We only found two studies from Western Europe comparing dogs and cats entering and leaving shelters. Both of these were based on questionnaires sent to dog and cat shelters in the UK. One study [25] covers the year 2009 and had responses from 55% of organisations, and the other [26] covered the year 2010 and had a response rate of 39%. The two studies found adoption rates of 77% and 75%, and euthanasia rates of 9% and 10% for dogs, while adoption rates of 90% and 77% and euthanasia rates of 5% and 13% were reported for cats. Compared to Denmark, slightly more dogs seemed to be euthanised, whereas the live release rate for cats in the UK seemed to be similar to what was found at the beginning of our study period in Denmark. However, the euthanasia rate for cats in Danish shelters at the end of the studied period was much higher than what was found in the UK, which can probably be explained by the specific Danish developments described above. A 2006 study of intake and output for cat shelters in Sweden (a neighbouring Scandinavian country) found that around 7400 cats entered shelters every year and less than 10% of these were euthanised [27]. Given that the population size of Sweden is nearly twice that of Denmark, the intake is similar to what we found around the start of the period studied, whereas the euthanasia rate was slightly lower. Overall, euthanasia rates for both dogs and cats seemed to be much lower in Western Europe compared to the USA and Australia.

The reviewed studies that covered developments over time, like our own study, saw a fall in the intake of dogs, while many saw an increase in the intake of cats. Similar to our findings, nearly all of the studies reported a higher euthanasia rate for cats than for dogs in shelters. Also in line with our findings, a report from the US [28] and a study from Taiwan [29] found a positive correlation between intake and euthanasia.

### 4.4. Limitations

Our study has a number of limitations; for example, the data from the shelters used in the current study were obtained from many different sources and using different types of recording practices. There is a risk that these practices varied among the shelters. As mentioned above, shelter managers were interviewed to resolve any issues caused by discrepancies in their data-recording practices. The completeness of the data was almost 90% in 2017, but this could not be recorded for the previous years, as several of the shelters no longer existed and the data from such shelters could not be retrieved. The majority of these are likely to have been small shelters, but we could not determine the degree of completeness and this was likely to have been lower in the first years of the study period. This could have an impact on the estimated proportion of cats. However, the major cat organisations provided data for the entire study period. For example, including only data from the four largest shelters that provided data for the entire period from 2005 to 2017 for cats, resulted in LRR_cat,2005_ = 0.85 and an LRR_cat,2017_ = 0.65 instead of 0.89 and 0.71, respectively, and the intake of cats would still have more than tripled from 2083 to 8201. Therefore, the drivers of numbers included were the shelters providing data both in the beginning and the end of the study period. The reasons for the increase has not been elucidated, but may have been affected by the changed visitation procedures, the established hotline, increased awareness, increased communication via social media etc., but still the animals recorded in the shelters are considered a valid representation of their presence there. Furthermore, we believe that we have included the majority of the population, because the Danish shelter community is relatively small and coherent, so that shelters with a significant intake would not go unnoticed. 

The hotline for cats hit by vehicles has only existed since 2011, and the data are only an approximation of the level of the population of homeless and stray cats. However, the trend seems to be the opposite of that found in the shelters. We must also accept the uncertainty related to this estimate, particularly because advertisements for the hotline were more common in the beginning than at the end of the current reporting period. However, users may also have become accustomed to using the hotline, which would increase its use. Therefore, the proxy measure of the population of homeless and stray cats should be interpreted with caution.

## 5. Conclusions

Over the study period between 2004 and 2017, we found a low and slightly decreasing yearly intake of dogs, combined with a low but slightly increasing rate of dogs being euthanised in Danish shelters. In contrast to this, we found a major increase in the intake of cats to Danish shelters, combined with a much higher euthanasia rate that doubled over the same period. The difference between the dogs and cats moving in and out of Danish shelters reflects a difference in how the two species are kept and regulated in a wealthy northern country like Demark. While dogs are generally kept under control by the owner (i.e., by the use of leashes and fenced gardens), and are tightly regulated, cats are kept in less controlled and regulated ways, and therefore cat shelters may not on their own solve the problem of surplus cats. It is clear that cats are more difficult to control than dogs, which is also reflected in the international literature. However, economic analysis may contribute to a discussion of how to improve policy making for NGOs in charge of shelters.

## Supplementary Materials

The following are available online at https://www.mdpi.com/2076-2615/9/10/765/s1: Supplementary text S1. Cooperation agreement with shelters, Supplementary text S2. Correspondence with key cat organisations on their knowledge on the development from 2004-2017, Supplementary data S1. Dataset on calls for hotline about cats hit in traffic, Supplementary data S2. Anonymised raw data on the traffic of cats and dogs in and out of shelters.
