# Supplementary material for: Shelters Reflect but Cannot Solve Underlying Problems with Relinquished and Stray Animals—A Retrospective Study of Dogs and Cats Entering and Leaving Shelters in Denmark from 2004 to 2017
Source: Animals (Basel). 2019 Oct 5;9(10):765. doi: 10.3390/ani9100765 (PMC6826399; doi:10.3390/ani9100765)
Supplement: Supplementary file 1 [file animals-09-00765-s001.zip › SuppText1. Cooperation agreement.docx]

**Cooperation agreement**

This cooperation agreement is concluded between

Janne Barner Hanquist Jensen
Research Assistant, University of Copenhagen

(hereafter referred to as Part A)

and

[Name]
[Position, Animal Welfare Organisation]

(herafter referred to as Part B)

1. **Background**

1.1 The cooperation agreement contains the shared understanding of the parties with regard to the sharing of data to a project with the aim to map, highlight and discuss the development of the flow of dogs and cats in animal shelters in Denmark.

**2. Main conditions**

2.1 Part B contributes to the cooperation by submitting raw data on relinquished and adopted dogs and cats from animal shelters, and any necessary information on the creation of these data.

2.2 After completion of the project, is Part A, along with Peter Sandøe and Søren Saxmose Nielsen, justified to create multiple scientific articles based on found results. These articles will be published both internationally and nationally.

2.3 There will be no discussion on animal cruelty cases, shelter politics, euthanasia or specific events deriving from the cooperation with Part B in the final project and articles. Furthermore, data will be presented in a way which makes it not possible to derive anything about conditions on named shelters.

2.4 Part B will in connection to publication get access to the article drafts for revision and commenting with a deadline of three weeks prior to publication preview.

**3. Confidentiality**

3.1 Part A is not allowed to lease Confidential Information to a third party, with exception of Peter Sandøe and Søren Saxmose Nielsen, unless there is a legal obligation to do so, or use Confidential Information for other purposes than stated in this cooperation agreement.

3.2 In item 3.1 is “Confidential Information” defined as any information shared during the compilation of Part A’s project, with the exception of information which is commonly available to or known by the public without it being a result of a Part’s unauthorized disclosure of the information.

3.3 The name of Part B and shelter staff will be left out, unless otherwise agreed, in connection to publication.

For Part A For Part B
