# Supplementary material for: Shelters Reflect but Cannot Solve Underlying Problems with Relinquished and Stray Animals—A Retrospective Study of Dogs and Cats Entering and Leaving Shelters in Denmark from 2004 to 2017
Source: Animals (Basel). 2019 Oct 5;9(10):765. doi: 10.3390/ani9100765 (PMC6826399; doi:10.3390/ani9100765)
Supplement: Supplementary file 1 [file animals-09-00765-s001.zip › SuppText2. Correspondence with Cat Welfare Organizations.docx]

**Correspondence with key cat organisations on their knowledge on the development from 2004-2017**

Topic: please, can you help me understand..?

Dear [recipient name]

My colleague Søren Saxmose Nielsen and I are working on a project on animal shelters financed by the Danish Veterinary and Food Administration. The project is based on Janne Jensen’s Master Thesis, of which you probably have heard of.

I am currently in the process of combining the results in a presentation and an article. In relation to this, I am in need of a little help from you and others with knowledge on animal shelters to answer some questions.

The most noteworthy are the results in relation to the development of cats entering Danish shelters. Please view the attached figures with a capacity-coverage of 90% of Danish shelters in the time period of 2004-2017.

Figure 1 clearly demonstrates that a threefold increase in the intake of cats is seen. The increase occurs especially from 2004-2008 and from 2012-2015. As depicted in Figure 2, the increase is mainly due to an increase in the number of stray cats, while the number of owner relinquished cats does not change much.

Simultaneously, we have no reasons to believe that the increase in intake is due to there being more stray cats due to the development in number of reported cats injured in traffic (Figure 1).

Thus, there are seemingly two possible explanations for the development in the mentioned periods: 1) that the shelter-capacity has increased, or 2) that there has been a change in the mentality of the relinquishers of stray cats towards cats

My questions are:

1. What happens during the two periods?
2. Can you suggest others I need to get in touch with to find the answer to 1)?

If more convenient, I would like to call you by phone to discuss this matter.

Kind regards,
Peter

**Response 1 – Monday May 13^th^, 2019**

Dear Peter,

Thank you, for your e-mail.

The launch of [an animal] emergency hotline on August 17^th^, 2011, has resulted in an increase from approximately 4,000 relinquished cats to more than 6,000 relinquished cats on a yearly basis.

In the period 2005-2008, collaboration with a number of new animal shelters was established. […]. This has probably resulted in more relinquished cats, as the volunteers could easily drop-off the cats at a nearby shelter rather than having to travel far.

[…]

Let me know, if you are in need of further answers.

Best regards,

[Respondent 1]

**Response 2 – Monday May 13^th^, 2019**

Hi Peter.

In the light of your enquiry, we have had a dialogue [at our shelter] on the reasons for the increase. We believe that several causal factors exist.

The increase [in use] of Social Media and increase in promotion of the cats on the animal shelters can be a contributing factor in the decision process on adoption of a shelter cat. This releases space to relinquished cats simply because the process of rehoming is faster.

Additionally, several campaigns on television, in the papers and on the Social Media urging [the public] to take responsibility for stray and free-roaming cats has taken place. Furthermore, the animal shelters have expanded in both numbers (more small shelters establishes) and capacity (existing shelters grow larger), which makes it easier for people to relinquish a newly found cat to a shelter or foster home for kittens. In any case, it seems that the sense of responsibility have increased and the belief that “cats can take care of themselves” decreases.

The Kitten Act may have contributed as well […]. The legal impediment that people can no longer hand off a kitten less than 12 weeks of age, may be a cause of dumping more kittens in boxes or alike out of desperation.

Kind regards,

[Respondent 2]

**Response 3 – Wednesday May 15^th^, 2019**

Dear Peter!

Nice to hear from you – it sounds like you are continuously working on interesting projects. I am doing great. […..]

I have looked at your questions, and I believe that the explanation relates to a number of things:

From when I was first employed at [the animal shelter] in yearX, a major change has occurred in the perception of cats and their status. In 1995, approximately half of relinquished cats were euthanized, as the shelters had troubles finding new homes for them – not because they were difficult cats, but because they [the cats] were in excess, and adoptions could not keep up with the number of relinquishments.

We wanted this to change, but at the time only had our membership magazines and by being on our marks.

With the more widespread use of the internet, it became easier for people could find information. I think this was around year 2000 that things started moving. It became easier for shelters to reach out to people and create webpages […], where we could put appetizers, which resulted in an increased popularity about cats. Our arguments were that cats were easier pets than dogs. And it is clear that the downside to increasing popularity was a higher number of surplus cats. Although cats adopted from shelters were neutralized, the large population of the cats in private homes was not, keeping the production of kittens up. My cautious guess is that the majority of relinquished cats were not strays, but merely surplus cats that owners could not get rid of themselves, and therefore handed them over to the shelters as cats found in a bush, etc. This would fit the massive increase in 2004-2008.

From 2008-2012, there was still plenty of “stray” cats, but the cats kept increasing in popularity. This was especially felt at the shelters: at [our shelter] the euthanasia rate decreased from around 50% in 1995 to below 10% in 2012. I believe that especially the use of the internet, as well as Facebook, was a major contributing factor.

In 2014, the number of relinquished cats peaked, which led the major animal welfare organizations to cooperate on increasing the focus on neutralization. The Cat Protection, Inge’s Home for Cats, Animal Protection Denmark, Danish Animal Protection Aarhus, the Danish Veterinary Association, The Animal Foundation, DOSO, The Danish Cat Register and Danish Cat Registration joined in a committee and invented The Cat’s Week (Week 39), where everyone went “all in” on increasing people’s awareness on neutralization. This has resulted in a small decrease in the number of “stray” cats, however, as it is expensive to neutralize, a lot of people still do not do it. Owners of male cats because it is not their problem. And owners of female cats because it is expensive to neutralize (and it is so nice having a litter of kittens). I believe the production is ongoing due to this. And as it is a low cost to relinquish [the cats] at a shelter, the sense of responsibility decreases on this issue as well, as people can just relinquish [the kittens] if they are not able to get rid of them themselves.

[…]

Kind regards,
[Respondent 3]

**Response 4 – Friday May 24^th^, 2019**

Dear Peter,

[…]

I have looked at the statistics on captured cats [by our animal organization], from 2008-2018.

It is hard to explain what causes the fluctuating number. However, it is worth noticing the number of cases from the Municipality X, whom which we have cooperated with since 1980. Copenhagen is pretty stable concerning the number of cases.

From 2008, the number of cases in Municipality X was 201 and the number of cases 123 was in 2018. However, in the year of 2010, the number of cases increased to 240 in Municipality X. But only this one year.

In 2012, [due to the Animal Shelter having economic and management issues, the Animal Shelter had to decrease activities]. […] this resulted in the closing of three shelters.

The beginning of the Financial Crisis in 2009 resulted in a doubling of forced sales, which caused that a lot of cats were abandoned in the residential areas.

Simultaneously, the municipalities had to save money on the [agreements made on capturing of cats], which had a large effect on the number of captures.

From 2009 and 2010, our number of cases increases with 400, and from 2012 to 2013, the number of cases decreases with 225 cases.

In 2010, we had the largest number of cases; 2.569 requisitions in total.

All our staff handling stray cats believe that the cats are getting tamer, but it also means a lot whether the shelters have room for these cats, and if there were more time and resources to socialize cats, more could be saved and adopted.

The number of releases [of previously captured cats] is pretty stable, but the number increases slightly from 2013-2016. In 2010, where we had a lot of captures, the rate of releases [of previously captured cats] decrease to 8.2.

The spread of Smartphones with users on Social Media has also connected friends of cats across the country, which means a lot for how people help each other to help cats.

The larger cities leads in [the rate of] awareness in relation to the animals, and when I speak to our staff handling cats in the rural areas on Western Zealand, Southern Zealand and Northern Zealand it is clear that they are on their way too, though it takes some time to better the status of the cats.

[…]

Kind regards,
[Respondent 4]
